# The diagnostic accuracy of the hand-held Raman spectrometer for the identification of anti-malarial drugs

**DOI:** 10.1186/s12936-016-1212-y

**Published:** 2016-03-15

**Authors:** Benjamin J. Visser, Sophia G. de Vries, Emmanuel B. Bache, Janneke Meerveld-Gerrits, Daniëlle Kroon, Jimmy Boersma, Selidji T. Agnandji, Michèle van Vugt, Martin P. Grobusch

**Affiliations:** Department of Infectious Diseases, Division of Internal Medicine, Center of Tropical Medicine and Travel Medicine, Academic Medical Center, University of Amsterdam, Meibergdreef 9, PO Box 22700, 1100 DE Amsterdam, The Netherlands; Centre de Recherches de Médicales de Lambaréné (CERMEL), Albert Schweitzer Hospital, Lambaréné, Gabon; Institute of Tropical Medicine, University of Tübingen, Tübingen, Germany; Pharmaceutical Technology and Biopharmacy, Utrecht University, Utrecht, The Netherlands

**Keywords:** Artemisinin combination therapy (ACT), Central Africa, Counterfeit, Drug quality, Falsified, Field survey, Gabon, Malaria, MEDQUARG, NanoRam^®^, Sensitivity, Specificity, Raman spectroscopy

## Abstract

**Background:**

There is a need for accurate and field-applicable instruments for the evaluation of the quality of anti-malarial drugs. The aim of this study was to determine the diagnostic accuracy of the NanoRam^®^, a handheld Raman spectrometer (RS), to identify anti-malarial drugs.

**Methods:**

In total, 289 anti-malarial drugs collected in a randomized field survey in Gabon were evaluated. The samples were compared with authentic products as supplied by the official manufacturer. To determine the sensitivity and specificity of the handheld NanoRam^®^ spectrometer in the identification of anti-malarial drugs, a two-gate reversed-flow design was applied. The standards for reporting of diagnostic accuracy studies (STARD) were followed. The index test was the handheld RS. The reference test standards were thin layer chromatography and high performance liquid chromatography with ultraviolet photo diode array detection.

**Results:**

The sensitivity [95 % confidence interval (95 % CI)] and specificity of the RS to correctly identify an anti-malarial drug were 100 % (95 % CI 94.9–100 %) and 96 % (95 % CI 92.3–99.0 %), respectively. The RS could not differentiate between different batches of the same product or different manufacturers of the same product. Intra-observer agreement for 289 samples was 100 %. The average time to conduct the RS was 15 s per sample compared to 45 min per sample for TLC.

**Conclusion:**

The handheld RS holds promise as an easy-to-use, quick and field-applicable instrument for the evaluation of quality of anti-malarial drugs, potentially empowering pharmacists, drug inspectors and medical regulatory authorities.

*Trial registration* NTR4341 (Dutch Trial Registry)

**Electronic supplementary material:**

The online version of this article (doi:10.1186/s12936-016-1212-y) contains supplementary material, which is available to authorized users.

## Résumé

**Contexte:**

Il est nécessaire d’avoir des outils de terrain fiables pour l’évaluation de la qualité des médicaments antipaludiques. Le but de cette étude était de déterminer la précision diagnostique de l’outil NanoRam^®^, un spectromètre Raman portable (RS), pour identifier les principes actifs des antipaludiques.

**Méthodes:**

Au total, 289 médicaments antipaludiques collectés lors d’une enquête aléatoire au Gabon ont été évalués. Les échantillons ont été comparés à des produits authentiques, tel que fourni par le fabricant officiel. Afin de déterminer la sensibilité et la spécificité du spectromètre NanoRam^®^ portable dans l’identification des médicaments antipaludiques, une méthode à ‘*flux inversé à deux canaux*‘a été appliquée. Les normes pour la présentation des études de précision de diagnostic ont été respectées. Le test de l’indice était le spectromètre NanoRam^®^ portable. Les tests de référence étaient la chromatographie sur couche mince (TLC) et chromatographie liquide à haute performance avec détection à barrette de diodes photo à ultraviolets.

**Résultats:**

La sensibilité [95 % intervalle de confiance (IC 95 %)] et la spécificité de la RS pour identifier correctement un médicament antipaludique était de 100 % (IC à 95 % de 94,9 à 100 %) et 96 % (IC à 95 % de 92,3–99,0 %), respectivement. Le RS ne pouvait pas différencier entre différents lots du même produit ou provenant de différents fabricants. L’accord intra-observateur pour 289 échantillons était de 100 %. Le temps moyen pour effectuer la RS était de 15 secondes par échantillon par rapport à 45 min par échantillon pour la TLC.

**Conclusion:**

Le spectromètre portable Raman est prometteur comme instrument de mesure, facile à utiliser, rapide et facilement applicable sur le terrain pour l’évaluation de la qualité des médicaments antipaludiques, facilitant le travail des pharmaciens, des inspecteurs de qualité des médicaments et des autorités de réglementation médicale.

*Enregistrement de première instance* NTR4341 (Dutch Trial Registry)

## Background

Poor-quality, falsified (counterfeit), substandard and degraded anti-malarial drugs (for definitions see [1]) are important impediments to public health. Studies have highlighted the magnitude of the problem of poor quality anti-malarial drugs [2–8]. According to the latest World Health Organization’s (WHO) estimates, released in December 2015, there were 214 million cases of malaria in 2015 and 438,000 deaths [9]. Early diagnosis and treatment with appropriate anti-malarial drugs are pivotal [10]. The WHO recommends artemisinin-based combination therapy (ACT) for the treatment of uncomplicated falciparum malaria [11–13]. Negative consequences arise from the use of poor-quality anti-malarial drugs. Anti-malarials with low amounts of active pharmaceutical ingredients (APIs) may cause increased morbidity and mortality [14]. An estimated 122,350 [interquartile range (IQR): 91,577–154,736] under-five malaria deaths are associated with the use of poor-quality anti-malarials, representing 3.75 % (IQR: 2.81–4.75 %) of all under-five deaths in a sample of 39 countries [15]. However, these results should be interpreted with caution since there are large gaps in data prevalence of poor-quality anti-malarials [3, 16] and in case fatality rates [15]. As well, inadequately low concentrations of the APIs in poor-quality drugs may result in sub-therapeutic concentrations of the drug in patients, which may engender drug resistance [17]. Furthermore, the use of poor-quality anti-malarial drugs leads to significant socio-economic losses for patients and their kin, health-care systems and pharmaceutical industries producing the genuine product [18]. Societies can lose confidence in a pharmaceutical brand, drugs, pharmacies, and health-care providers [2].

The spread of poor-quality anti-malarial drugs increases the need for rapid detection of such medicines throughout the supply chain. The paucity of quality assurance laboratories in Africa with appropriate analytical capabilities (in terms of personnel and equipment) slows down the fight against poor-quality medicines. Several screening methods have been used, or have been investigated for use in screening drug quality in low-resource settings. The aim of a screening tool in drug quality surveys is to minimize the number of anti-malarial drugs that will undergo reference-standard testing in a (resource-limited) laboratory. Furthermore, the ideal screening tool should be low-cost, easy-to-use (not requiring specialist training), sustainable (especially in humid and hot climates) and easy and affordable to be repaired (locally). Ideally, the integrity of the sample should be sustained. Moreover, the total cost of identifying a conspicuous sample should be economically balanced in relation to the screening survey as a whole. Semi-quantitative thin layer chromatography, which was further developed by the Global Pharma Health Fund (GPHF), “the Minilab^®^”, has been available since the past couple of years [19, 20]. This technique is applicable in the field and relatively cheap (approx. 4500€ in 2014) but requires training, the use of chemicals, and is labour-intensive [21]. It can be used to identify the API and can therefore be used as a screening tool to detect falsified medicines. It is, however, a semi-quantitative method and results should, therefore, be confirmed using more accurate methods, such as high-performance liquid chromatography (HPLC) [1].

Non-invasive (non-destructive) methods as a screening and identification tool of APIs have recently gained more scientific interest [22, 23]. A recent report evaluated the TruScan^®^ Raman device (using inelastic light scattering) for testing pharmaceutical products in the field to detect falsified and substandard medicines [24]. Six pharmaceutical products were under investigation, including analgesics and antidiarrhoeal medicines. It was shown that it could detect falsified drugs, but was not able to discriminate between good-quality and sub-standard drugs. The possibilities of Raman spectroscopy as a fast and reliable screening method for the detection of falsified artesunate tablets were investigated before, with the Renishaw System-1000^®^ spectrometer (Wotton-under-Edge, UK), connected to a conventional light microscope. The method was able to distinguish between genuine and falsified artesunate and to identify the composition of the falsified tablets [25]. It was demonstrated that the results of the Raman spectroscopy method were in agreement with those of colourimetric tests and of liquid chromatography-mass spectrometry of artesunate. The present study aims to explore the ability of Raman spectroscopy (RS) performed by the NanoRam^®^ handheld device to identify anti-malarial drugs and to differentiate between similar anti-malarial drugs. The diagnostic accuracy (sensitivity and specificity) of RS was determined, compared to thin layer chromatography (TLC).

## Methods

### Anti-malarial drugs tested

**Fig. 1:**
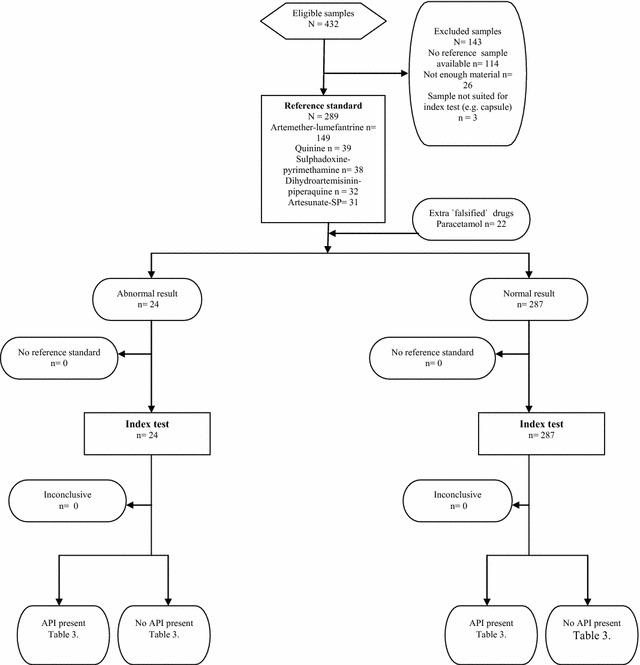
STARD Flow diagram: identification of anti-malarials. This diagram scheme is found at http://www.consort-statement.org/flow_test.pdf

The samples used in this pilot study were collected in a randomized field survey of anti-malarial drugs in Gabonese pharmacies [21], following the medicine quality assessment reporting guidelines (MEDQUARG) [1]. The randomized field survey was registered in advance (Trial registration number: NTR4341 [26]). Also, the costs of the present study are reported [27] (Additional file 1). Scientific clearance for the field survey was obtained from the Scientific Review Committee (SRC) of the Centre de Recherches de Médicales de Lambaréné (CERMEL), Albert Schweitzer Hospital (SRC number: 2013.11). The Ethical Committee of CERMEL decided that ethical approval of the field survey study was not required, as the study is a health care quality assurance study, no humans having been subjected to observation or intervention. The detailed methods were described earlier [21, 26]. In brief, anti-malarial drugs were collected by local fieldworkers from randomly selected pharmacies. In total 432 samples were collected, of which 289 were tested in the present study. Not all samples could be tested with the handheld Raman spectrometer (RS) because no reference standards of all the anti-malarial drugs could be obtained from the producing pharmaceutical companies; or the dosage form was not suitable for the handheld RS (e.g., capsule instead of tablet or liquid form); or no material was left from the collected sample (Fig. 1). The following five anti-malarial drugs were evaluated: artemether–lumefantrine (AL, n = 150), quinine (Q, n = 40), sulfadoxine–pyrimethamine (SP, n = 38), dihydroartemisinin–piperaquine (DHA–PQ, n = 32), and artesunate-SP (AS–SP, n = 31). These anti-malarials were produced by multiple pharmaceutical companies (Table 1). For analytical purposes, paracetamol (also known as acetaminophen) 500 mg [collected in the pharmacy of the Academic Medical Center (AMC)] was included since it is readily available and would be useful for studying the ability of the handheld RS to differentiate between different products.

**Table 1:** Summary of formulations tested with the brand name, dose, dosage form and manufacturer

Pharmaceutical product	Generic name and dose	Manufacturer or distributor
Arsiquinoforme^®^	Quinine tablet 250 mg	Sanofi Aventis
Artediam^®^	Artesunate–amodiaquine tablet 100/300 mg	Adams Pharmaceutical (Anhui) Co., Ltd
Artefan^®^	Artemether–lumefantrine tablet 20/120 mg	Ajanta Pharma Ltd, India
	Artemether–lumefantrine tablet 80/480 mg	
Artim^®^	Artemether–lumefantrine tablet 40/240 mg	Twight Litaka Pharma Limited Ltd, India
Artiz (Forte)^®^	Artemether–lumefantrine tablet 40/240 mg	Alice Pharma Pvt Ltd, India
	Artemether–lumefantrine tablet 20/200 mg	
Bimalaril^®^	Artemether–lumefantrine tablet 80/480 mg	Bengba Pharmaceutical factory, China
Coartem^®^	Artemether–lumefantrine tablet 20/120 mg	Novartis Pharma, Netherlands
Co-artemax^®^	Dihydroartemisinin–piperaquine tablet 40/320 mg	GA Pharma, Greece
Cofantrine^®^	Artemether–lumefantrine tablet 20/120 mg	EGR Pharma, Luxembourg
	Artemether–lumefantrine tablet 80/470 mg	
Colart^®^	Artemether–lumefantrine tablet 20/120 mg	Glaxo Smith Kline Group
Combimal^®^	SP tablet 500/25 mg	Ajanta Pharma Ltd, Mauritius
Darte-Q^®^	Dihydroartemisinin–piperaquine tablet 40/320 mg	Gosun Pharma Corp, China
Duo-Cotecxin^®^	Dihydroartemisinin–piperaquine tablet 40/320 mg	Beijing Holley-Cotec Pharmaceutical Ltd, China
Falquin®	Quinine tablet 300 mg	Plethico pharmaceutical Ltd, India
Fansidar®	SP tablet 500/25 mg	F.Hoffmann La Roche Ltd, Switzerland
Lariam®	Mefloquine tablet 250 mg	F.Hoffmann La Roche Ltd, Switzerland
Lufanter®	Artemether–lumefantrine 20/120 mg	Bliss Gvs Pharma Ltd, India
	Artemether–lumefantrine tablet 80/480 mg	
Lumart®	Artemether–lumefantrine tablet 20/120 mg	Cipla Ltd, India
	Artemether–lumefantrine tablet 40/240 mg	
Malacur®	Dihydroartemisinin–piperaquine tablet 40/320 mg	Elder pharmaceuticals LTD, India
Maloxine®	SP tablet 500/25 mg	Gracure Pharmaceuticals Ltd, India
Mephaquin®	Mefloquine tablet 250 mg	Mepha Ltd, Switzerland
Paracetamol	Paracetamol tablet 500 mg	Actavis/Allergan, Netherlands
P-Alaxin®	Dihydroartemisinin–piperaquine tablet 40/320 mg	Bliss Gvs Pharma Ltd, India
Quinimax®	Quinine 500 and 125 mg	Sanofi Aventis
R-Lume®	Artemether–lumefantrine tablet 80/480 mg	Impact Healthcare Pvt Ltd, India
Surquina®	Quinine tablet 250 mg	Laboratoire Innotech International, France

### Reference test: thin-layer chromatography

Detailed reference test methods have been described before [16]. GPHF Minilab^®^ [20] was used to run semi-quantitative thin-layer chromatography (TLC) and disintegration testing to measure the concentration of APIs. The samples failing the TLC test were analysed by HPLC, for confirmation. The MiniLab^®^ protocols award products a ‘pass’ for TLC if 80 % or more of the labelled active ingredient(s) is present. For fixed-dose combinations (e.g., AL) and SP, ‘pass’ was awarded only if both active ingredients met this standard. TLC is an accepted method to assess the quality of drugs [28–30].

### Index test

The present study was conducted in June and July 2015 at the AMC, University of Amsterdam, The Netherlands. All the tests were carried out in similar environmental conditions of light (±500 Lux), humidity (70 %) and temperature (±21 °C). The index test was conducted by BJV, the reference test by BJV and JMG (TLC) and H. Kaur, London School of Tropical Medicine and Hygiene, UK (HPLC). Inter-observer agreement was calculated for the TLC test, but not for the HPLC and index test. The researcher conducting the index test was not blinded to the outcome of the reference test. The index test and reference test are seldom perfect; therefore, the test reproducibility was calculated using the kappa, the intra-observer agreement [31]. All the index tests were conducted in duplicate.

### Index test: Raman spectrometry-technique

**Fig. 2:**
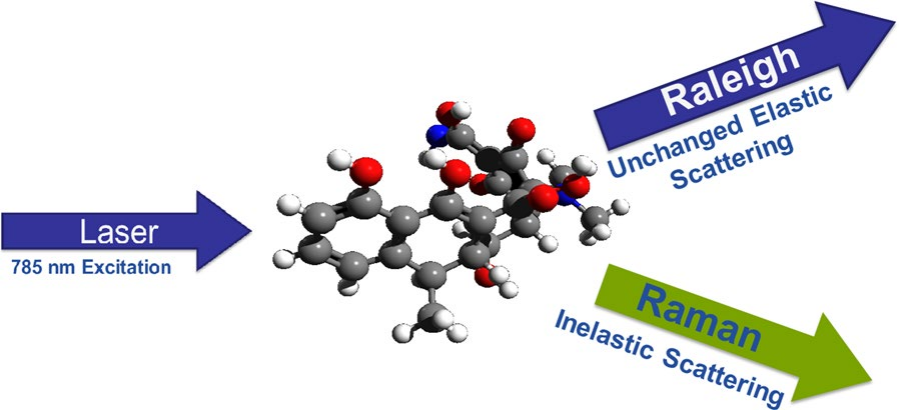
Raman spectroscopy technique “inelastic light scattering”

Raman spectrometry (inelastic light scattering or “Raman scattering”) is a light-scattering process in which the sample under examination is irradiated with intense monochromatic light; the light scattered from the specimen is analysed for frequency shifts (Fig. 2) [32]. This technique can be used to observe vibrational, rotational, and other low-frequency modes in a material [33]. Raman spectrometry is particularly sensitive to non-polar bonds (e.g., C–C single or multiple bonds) and less sensitive to polar bonds [22]. In chemistry, it is frequently used to provide a “fingerprint” by which a molecule can be identified [34].

### Index test: equipment

**Fig. 3:**
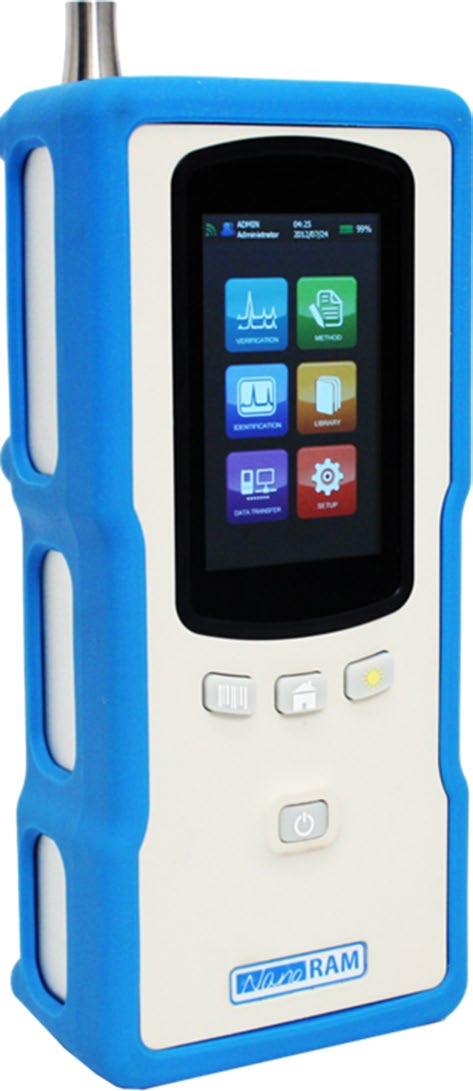
The NanoRam^®^ handheld Raman spectrometer

The NanoRam^®^ hand-held RS was used in this study (Fig. 3). The device (model: BWS456, Serial Number: SAMLNA, manufactured in March 2014) was rented from B&W Tek, Inc. 19 Shea Way, Newark, Delaware, USA, 19713). The NanoRam^®^ is a hand-held Raman instrument for non-destructive identification and verification of materials such as APIs, excipients, intermediates and finished products (see Additional file 2 for specifications). The use of the device does not require any training, and handling can be managed by untrained non-specialist users. An advantage of this technique is that it can analyse drugs through transparent containers (blisters), maintaining the integrity of the sample. Raman spectroscopy is an approved method by the U.S. and European Pharmacopoeia [35], as well as the Pharmacopoeia of the People’s Republic of China [36].

### Index test: factory calibration of the NanoRam^®^

The device used had been calibrated according to B&W Tek, Inc. calibration procedures to a Raman shift accuracy of ±4 cm^−1^. Standard calibration procedures included the creation of a calibration set using B&W Tek, INc. SCL series Spectral Calibration Lamps (Mercury and Argon) and Polystyrene Calibration Cap. SCL 100 series Light Sources have a wavelength accuracy of ±0.1 nm. The spectral line data for Mercury (Hg) and Argon (Ar) are referenced in “Wavelength and transition probabilities for atomic ions, part 1—wavelength”, a publication of the National Standards Reference System, National Institute of Standards and Technology, U.S. Department of Commerce [37]. The calibration was conducted on 30-7-2014 and is valid for a year.

### Index test: establishment of a spectral reference library

The NanoRam^®^ spectral device includes a library of 110 API’s. However, no spectral references for anti-malarial drugs were provided with the NanoRam. Therefore, the spectra of different anti-malarial drugs which are known to be of good quality and which exhibit the variation (manufacturer, batch, crystal modification, particle size) typical of the anti-malarial to be analysed, were created using the instrument’s built-in method without modification. For each reference spectrum, 20 runs (scans) are necessary. This spectrum or signature is used to compare with spectra of other test samples. In this study, only samples provided by the producing pharmaceutical company were considered as ‘reference samples’. Libraries for different anti-malarial drugs and dosages were created (AL, DHA–PQ, Q, AS–SP, SP; for example libraries see Additional file 3). Match-or-fail comparison decisions were performed by the built-in software. Tablets (remaining inside the transparent blisters) were placed in front of the laser. Raman bands [Wave number (cm^−1^)] can be assigned to corresponding functional groups. The Raman characteristic frequencies of organic molecules and inorganic compounds have been described elsewhere [38–40]. The main outcomes of the test of a sample are a ‘match or fail’ result. This match-or-fail outcome is based on the p value automatically calculated by the handheld RS between the comparator (the reference spectrum) and the spectra of the sample under investigation.

### Study design and statistical methods

Where appropriate, the standards for reporting of diagnostic accuracy studies (STARD) were followed [41] (Additional file 4). To determine the sensitivity and specificity of the handheld device in the identification of anti-malarial drugs, a two-gate reversed-flow design was applied [42]. The index test (Raman spectroscopy performed by the NanoRam^®^ handheld spectrometer) and reference standard (thin layer chromatography and high performance liquid chromatography with ultraviolet photo diode array detection) were performed in reverse order. Reversing the order in which the index test and reference standard are conducted will not change the estimates of diagnostic accuracy. This type of design was chosen because of practical reasons since the samples were evaluated in another study already [21], and the RS was not yet available in our laboratory. In the preferable one-gate design, cases and controls are sampled from the same pool (anti-malarial drug collection); this was however not possible due to a lack of ‘cases’ (falsified drugs), thus a two-gate design was necessary (see below). To achieve sufficient power for the diagnostic accuracy calculations, the sample size was calculated as described in the sample size methods by Flahault et al. [43]. When the prevalence of poor quality drugs is less than 50 %, two assumptions ought to be made: the expected specificity (or sensitivity) values of the new diagnostic test, and the minimum acceptable lower confidence limit. For the NanoRam^®^, two studies showed high sensitivity and specificity, but exact numbers were not reported [24, 25].

Assuming that the specificity will be high, at the minimal acceptable confidence limit of 0.7 (together with the required probability which was set here at 0.95 that this limit is not violated), the minimum number of falsified or poor-quality samples required is 24 [43]. The prevalence of poor-quality drugs in Gabon is 0.5 % [(95 % CI 0.08–1.84 %) [21]. Using the prevalence of 0.5 %, the number of controls would be 199, using the equation Ncontrols = Ncases × [(1 − Prevalance)/Prevalence]. The controls come from the same pool as the cases, thus the definite sample size is 223. Since the reference test was already conducted before the index test, it was known that only two samples were of poor quality [21]. Therefore, 24 − 2 = 22 extra controls were added (“falsified anti-malarial drugs”: in this model paracetamol 500 mg was used). This two-gate model is an accepted design in the early investigation of a new diagnostic test [42], it has, however, been criticized for leading to inflated estimates of accuracy. Paracetamol was used since this product has been sold frequently as being an anti-malarial drug in South East Asia [44] and is readily available.

Data was analysed using SPSS (Version 21.0, IBM, NY, USA). The results of each diagnostic test of every sample were recorded in Excel and SPSS. The diagnostic accuracy was defined by sensitivity and specificity with 95 % confidence intervals (CIs), which were computed as by standard literature [45, 46]. A cross tabulation of the index text results by the results of the reference test was conducted.

## Results

In this study, a total of 289 anti-malarial drugs were tested (Fig. 1). The index test was conducted within 8 months after the reference test. All samples were tested while remaining in their transparent blisters, except for DHA–PQ tablets (n = 32, 11 %): the blisters were transparent but the tablets have a thick blue coating resulting in a lack of penetration of the laser. These tablets were broken into half with a pill splitter, and subsequently tested. On average, a reference spectrum of an anti-malarial drug (20 runs) could be created within 3.5 (min–max: 3–6) minutes. The average time to conduct a match/fail analysis for one sample was 15: 10 s of entering data (name and sample number of the drug under investigation) and 5 s for scanning the drug sample and obtaining a match/fail result. In comparison, the semi-quantitative layer chromatography technique used by the GPHF MiniLab^®^ costs on average approximately 45 min processing time per sample.

### Precision test of the NanoRam spectrometer

**Fig. 4:**
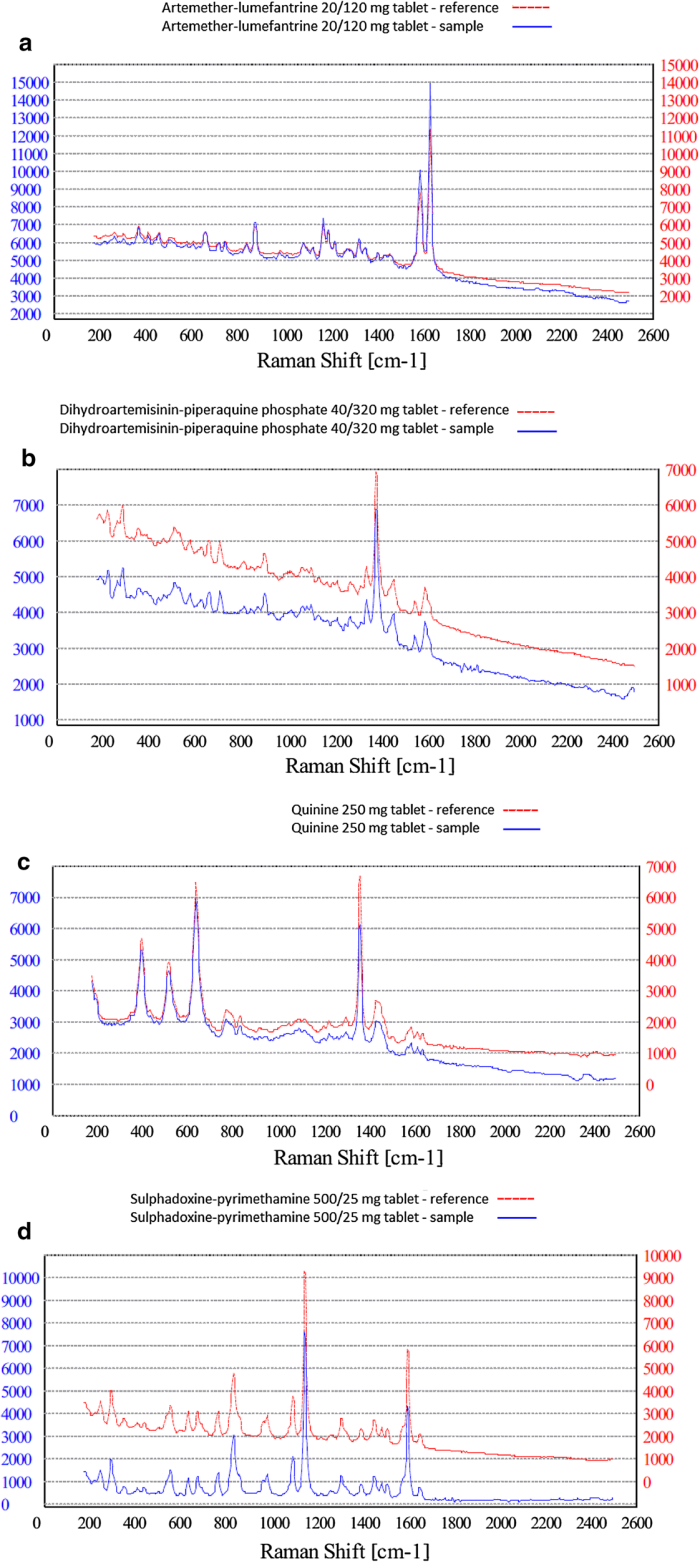
Raman spectroscopy precision test results for **a** artemether lumefantrine 20/120 mg. **b** Dihydroartemisinin–piperaquine **c** quinine sulphate **d** sulfadoxine–pyrimethamine

**Fig. 5:**
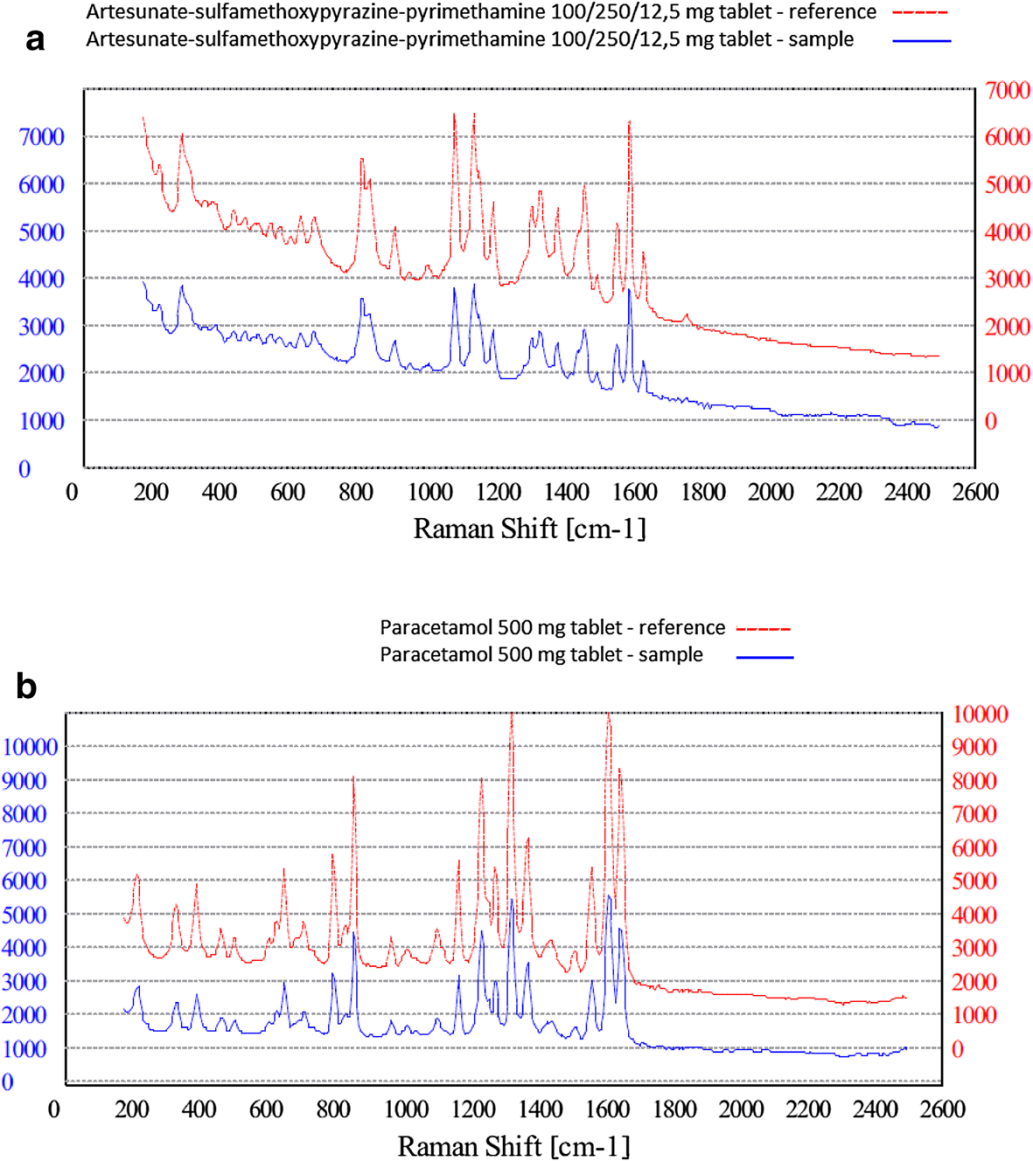
Raman spectroscopy precision test results for **a** Artesunate-SP, **b** Paracetamol

To investigate the capability of the NanoRam spectrometer to produce reproducible results, a match/fail analysis using a reference tablet was compared with the reference spectrum of the same sample. All the precision tests were conducted under the same circumstances and in duplicate. AL, DHA–PQ, Q, SP, AS–SP and paracetamol were utilized in different strengths. Table 2 depicts the results of the precision tests, Figs. 4a–d and 5a, b show examples of the precision test. Important to note is that the results of the match/fail scans are adjusted for the concentration of API in the tablet. For example, a match/fail test of AL 40/240 mg tablet matched the NanoRam’s reference spectrum of AL 20/120 mg tablet. In strict sense, this should be a “fail”. However, the NanoRam spectrometer only measures the concentration of the API in the tablet, and does not take into account tablet size or total weight, which may differ between different strengths of the same drug. In this particular case, the 40/240 mg dosage contained the same concentration of API as the 20/120 mg tablet, but the tablet was twice as big.

**Table 2:** Precision tests

Sample	Number of reference samples	Number of samples matching signature	Percentage of match (%)
Artemether–lumefantrine 20/120, 40/240, 80/480 mg	72	72	100
Quinine sulphate 300 mg	35	35	100
Dihydroartemisinin–piperaquine 40/320 mg	12	12	100
Artesunate–sulfamethoxypyrazine–pyrimethamine 100/250/12,5 mg	14	14	100
Paracetamol 500 mg	48	48	100

### Diagnostic accuracy of the RS

**Fig. 6:**
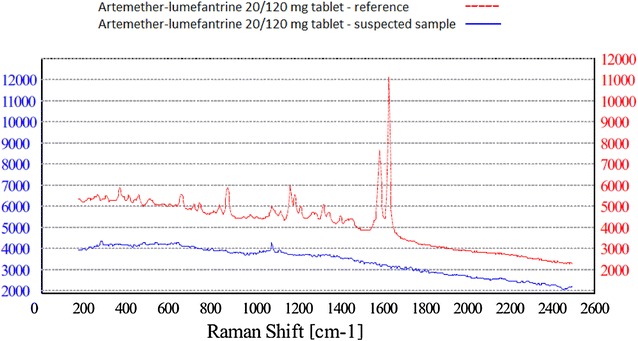
Raman spectroscopy of suspected artemether–lumefantrine sample

**Fig. 7:**
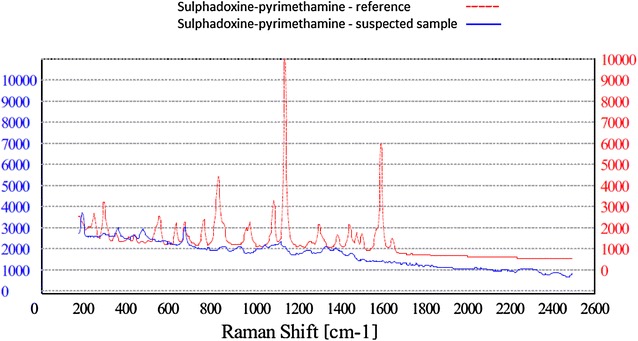
Raman spectroscopy of suspected SP sample. Sulphadoxine–pyrimethamine suspected sample containing only have the dose

To investigate the sensitivity and specificity of the device, different comparison scenarios to assess the ability of the RS to discriminate between different samples were evaluated. First, the diagnostic accuracy of the RS to correctly identify the API in the anti-malarial drug was determined. The 289 anti-malarial drugs plus additional fakes (22 paracetamol 500 mg tablets) were analysed with the RS. The sensitivity and specificity were 100 % (95 % CI 94.9–100 %) and 96.2 % (95 % CI 92.3–99.0 %) respectively (Table 3). The falsified AL sample was correctly identified as having no API inside the tablets (Fig. 6). The suspected SP sample which failed the TLC test, was demonstrated with HPLC to contain the stated API, but the amount was approximately halve of the dose. The API was not identified by the NanoRam and thus “failed” the test (Fig. 7). However, the device suggested the API SP as closely related to the generated spectrum. 11 samples of SP “failed” the RS test, but passed the reference test. These 11 SP samples failed the RS test again in the subsequent round. The device suggested the API SP as closely related to the generated spectrum. Difficulty in using Raman spectroscopy in identifying SP has been previously reported [47]. A reason for this is the high fluorescence of SP itself. The 22 paracetamol tablets were all correctly identified as being a non-malarial drug.

**Table 3:** 2 × 2 Table Raman spectroscopy vs. thin-layer chromotography

	Falsified/cases (fail TLC)	Non-falsified/controls (pass TLC)	Total
Fail RS	24 (TP)	11 (FP)	35
Pass RS	0 (FN)	276 (TN)	276
Total	24	287	311

*TP* true positive, *FP* false positive, *FN* false negative, *TN* true negative, *RS* Raman spectroscopy, *TLC* thin-layer chromotography

### Comparison of different lots of the same product

AL, Q, SP were used in this test. One batch of AL (Coartem, Novartis, Batch: X1639) was used to generate a Raman reference spectrum. This reference spectrum was compared to samples of the same batch (X1639), and 35 samples of 15 selected other batches of the same manufacturer Novartis. All samples matched to the comparator batch. For SP and Q, no differences between different lots of the same product could be identified.

### Comparison of similar products from different manufacturers

The capacity of the RS to discriminate between similar products from different pharmaceutical companies was also investigated (Table 1). The RS could not discriminate between products with the same API from different manufacturers.

## Discussion

This study represents the first evaluation of the diagnostic accuracy of the NanoRam^®^ spectrometer to identify and discriminate between anti-malarial drugs. The results suggest that the handheld NanoRam^®^ spectrometer has a high sensitivity and specificity to identify anti-malarial drugs. All falsified anti-malarials were detected by the RS. Some samples (n = 11, only SP) contained the stated API, but still failed the test (“false positives”). The results suggest that the NanoRam^®^ can be used to screen large quantities of anti-malarial drugs in a fast and non-destructive way, but caution is required when screening SP tablets. Minimal training was required and agreement between the tests was 100 %. Reference standards for the NanoRam^®^ are not provided with the device but can be easily created and shared between users around the globe.

### Strengths and limitations

The major strength of the present study is the large number of different random collected anti-malarial samples, which enabled us to investigate and create different reference spectra for the anti-malarial drugs. Another strength is that the sample size was sufficient to calculate the diagnostic accuracy. A major limitation of this study is that the reference standard is a semi-quantitative method, and that the sensitivity of the reference method is limited [48]. This means that false negatives might be present. The probability of false positives has been reduced, by analysing the samples failing TLC also with HPLC. It was not possible to analyse all samples with HPLC because the funds for this study were insufficient (HPLC of all samples costs approximately 14,000€). An important next step for further research should be an evaluation of the diagnostic accuracy of the handheld RS compared to the reference standard HPLC. Another limitation of our study is that we had a number of missing reference samples and had to exclude 143 anti-malarial samples. Moreover, the number of ‘true’ falsified anti-malarials was small, and an extra case group (paracetamol tablets) had to be added, which is a source of bias and might have led to an overestimation of the diagnostic accuracy. Another potential source of bias is that the investigator could not be blinded for the results of the reference test; this risk is minimal because the device provides a ‘pass’ or ‘fail’ with a *p* value, leaving little room for interpretation errors. Lastly, a limitation of this study is that the ability of the handheld RS device to discriminate between different dosage strengths as an indicator of the quality of anti-malarial drugs was not investigated. The number of different samples with different dosages for this analysis was insufficient. The ability to differentiate between dosages is important since products with reduced APIs have been produced.

The handheld RS (NanoRam^®^) device is commercially available but expensive. However, it could be cost-effective to have a device as a medicine regulatory authorities since it can reduce the number of products that will undergo reference testing in specialized laboratories overseas, which is time-consuming and even more expensive.

## Conclusion

The results have demonstrated that Raman spectroscopy performed by the NanoRam^®^ has a high sensitivity and specificity to identify and discriminate between anti-malarial drugs. In future medicine quality surveys, a handheld RS, preferably in combination with reference methods such as HPLC, may become the preferred method to assess the quality of large quantities of anti-malarial drugs throughout the supply chain. Hence, a handheld RS can be a valuable tool in the ultimate goal of malaria elimination.

## Additional files

10.1186/s12936-016-1212-y The costs of this study.
10.1186/s12936-016-1212-y NanoRam^®^ product specifications.
10.1186/s12936-016-1212-y Library methods figures.
10.1186/s12936-016-1212-y STARD checklist.

## Abbreviations

ACT: artemisinin-based combination therapy
AL: artemether–lumefantrine
AMC: Academic Medical Center
API: active pharmaceutical ingredient
CERMEL: Centre de Recherches Médicales de Lambaréné
GPHF: The Global Pharma Health Fund
HPLC: high-performance liquid chromatography
INN: international non-proprietary name
MEDQUARG: medicine quality assessment reporting guidelines
RS: Raman spectrometer
SP: sulfadoxine–pyrimethamine
TLC: semi-quantitative thin-layer chromatography
USP: US Pharmacopeia
UV: ultraviolet-visible
WHO: World Health Organization
