# Supplementary material for: The diagnostic accuracy of the hand-held Raman spectrometer for the identification of anti-malarial drugs
Source: Malar J. 2016 Mar 15;15:160. doi: 10.1186/s12936-016-1212-y (PMC4791808; doi:10.1186/s12936-016-1212-y)
Supplement: Supplementary file 1 — 10.1186/s12936-016-1212-y The costs of this study. [file 12936_2016_1212_MOESM1_ESM.doc]

**Additional File 1:** Study costs

**Summary of research costs of the diagnostic accuracy study with NanoRam® handheld spectrometer (including chemical analysis, excluding personnel).**

| **Specification** | **Amount in CFA-franc*** | **Amount in euro (€ )** | **Percentage of total costs** |
| --- | --- | --- | --- |
| Antimalarial drugs collected in Gabon | 1,619,572 CFA | 2468,86 € | 24,3% |
| GPHF Minilab™ | n/a | 4491,40 € | 44,1% |
| HPLC analysis (LSHTM) | - | In kind contribution | - |
| Personnel costs | NS | NS | NS |
| Introduction & rental fee for the handheld NanoRam® spectrometer | n/a | 1500,00 € | 14,7% |
| Article-processing charge BMC Journal | n/a | 1720,00 € | 16,9% |
| ***Total costs*** |  | *10,180.26* | *100%* |

* CFA= Communautés Financières d'Afrique (1 € = 656 CFA) ; NS=not specified
