# Supplementary material for: The diagnostic accuracy of the hand-held Raman spectrometer for the identification of anti-malarial drugs
Source: Malar J. 2016 Mar 15;15:160. doi: 10.1186/s12936-016-1212-y (PMC4791808; doi:10.1186/s12936-016-1212-y)
Supplement: Supplementary file 2 — 10.1186/s12936-016-1212-y NanoRam® product specifications. [file 12936_2016_1212_MOESM2_ESM.doc]

**Additional File 2**: Technical specifications

| **Principle of operation** | **Raman spectroscopy (NanoRam®)** |
| --- | --- |
| Excitation Wavelength | 785nm |
| Laser Output Power | 300mW or below, software adjustable |
| Spectral Range | 176cm-1 to 2900cm-1 |
| Spectral Resolution | ~ 9cm-1 @ 912nm |
| Detector Type | TE Cooled Linear CCD Array |
| Display | High Brightness and High Resolution Touch Screen |
| Barcode Reader | Linear and 2D Standards |
| Software | NanoRam® OS (Embedded), NanoRam® ID (PC) |
| Data Formats | .txt, .csv, .spc |
| Connectivity | Ethernet, Wi-Fi |
| Battery | Rechargeable Li-ion, >4 hrs Operation |
| AC Adapter | Output: DC 12V, 2A Minimum |
| Weight | ~2.5 lbs (~1.2 kg) |
| Size | 8.8in x 3.9in x 2.0in (22cm x 10cm x 5cm) |
| Operating Temperature | -20°C to +40°C |
| Storage Temperature | -30°C to +60°C |
