# Supplementary material for: The diagnostic accuracy of the hand-held Raman spectrometer for the identification of anti-malarial drugs
Source: Malar J. 2016 Mar 15;15:160. doi: 10.1186/s12936-016-1212-y (PMC4791808; doi:10.1186/s12936-016-1212-y)
Supplement: Supplementary file 3 — 10.1186/s12936-016-1212-y Library methods figures. [file 12936_2016_1212_MOESM3_ESM.doc]

**Additional File 3:** Library methods

**Artemether-lumefantrine**

**
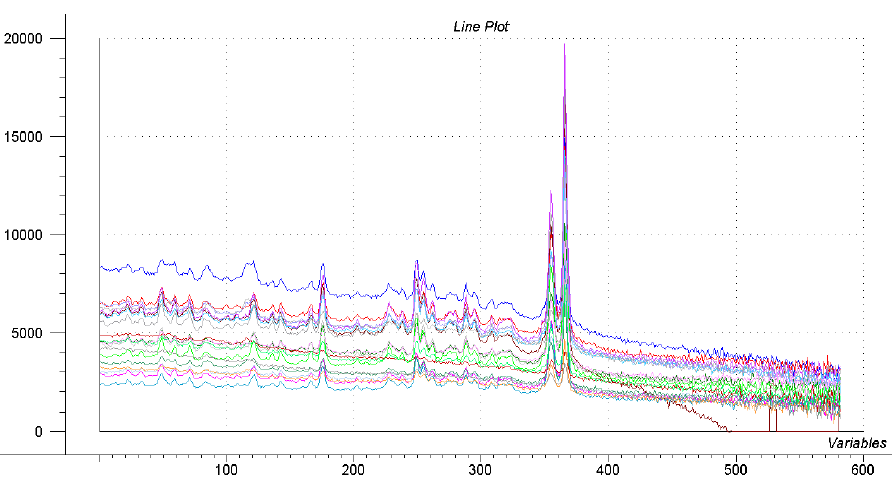
**

**Dihydroartemisinin-piperaquine**

**
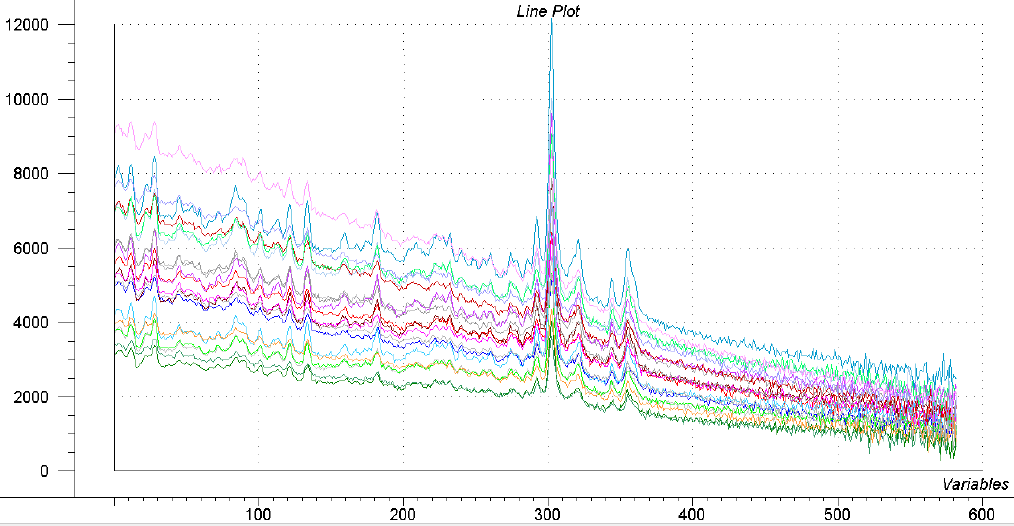
**

**SP**

**
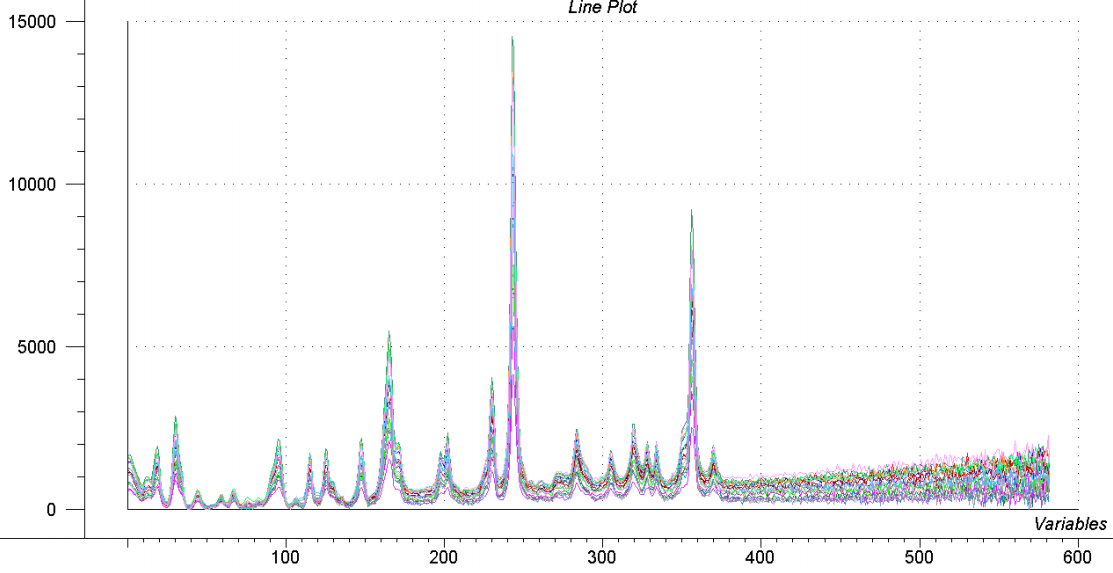
**

**Quinine**

**
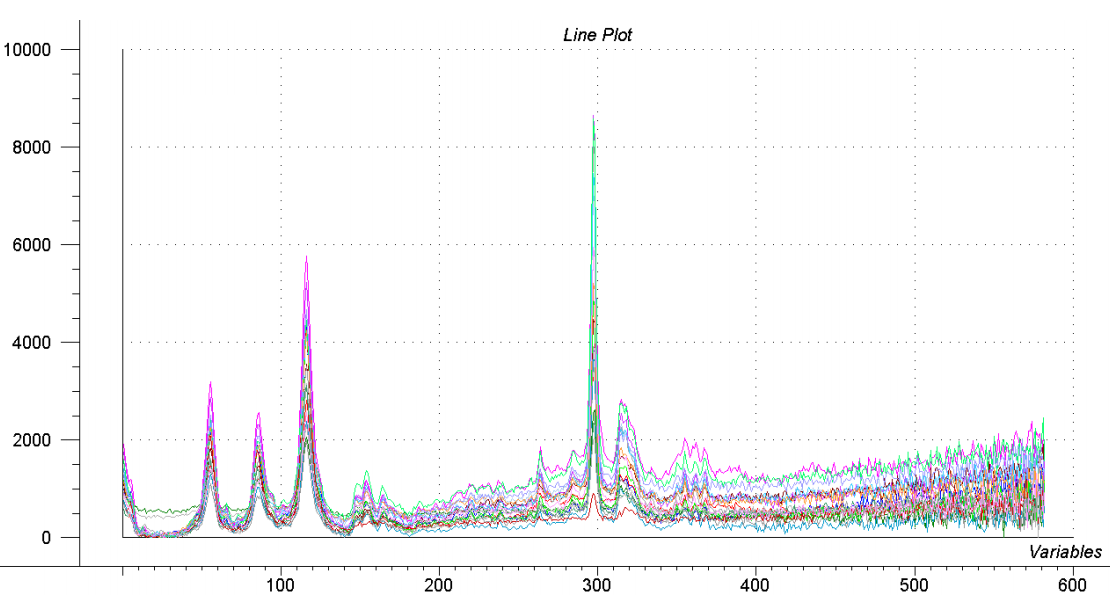
**

**Artesunate-SP**

**
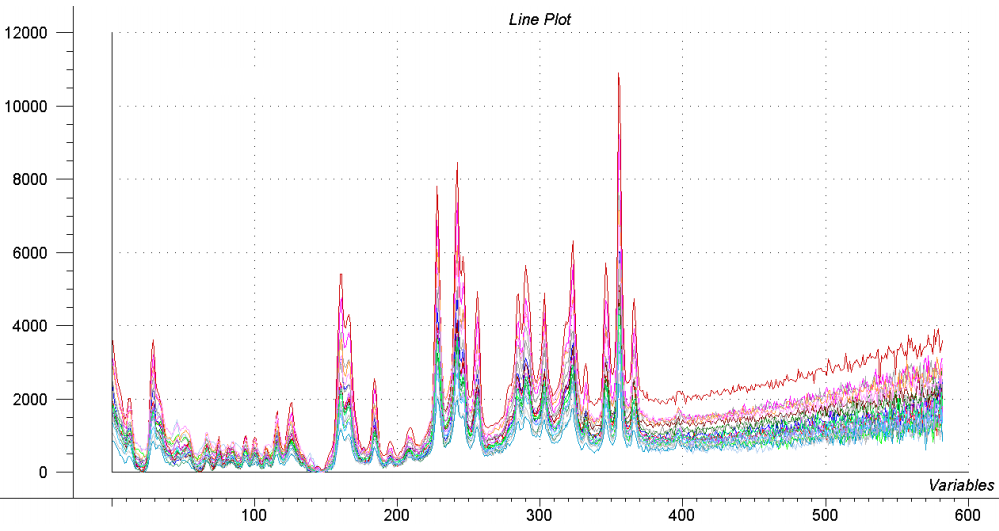
**
